# Use of Homogeneously-Sized Carbon Steel Ball Bearings to Study Microbially-Influenced Corrosion in Oil Field Samples

**DOI:** 10.3389/fmicb.2016.00351

**Published:** 2016-03-24

**Authors:** Gerrit Voordouw, Priyesh Menon, Tijan Pinnock, Mohita Sharma, Yin Shen, Amanda Venturelli, Johanna Voordouw, Aoife Sexton

**Affiliations:** ^1^Petroleum Microbiology Research Group, Department of Biological Sciences, University of CalgaryCalgary, AB, Canada; ^2^Oil Search LimitedBrisbane, QLD, Australia

**Keywords:** microbially influenced corrosion, weight loss, carbon steel, coupons, beads, sulfate-reducing bacteria, methanogens, acetogens

## Abstract

Microbially-influenced corrosion (MIC) contributes to the general corrosion rate (CR), which is typically measured with carbon steel coupons. Here we explore the use of carbon steel ball bearings, referred to as beads (55.0 ± 0.3 mg; Ø = 0.238 cm), for determining CRs. CRs for samples from an oil field in Oceania incubated with beads were determined by the weight loss method, using acid treatment to remove corrosion products. The release of ferrous and ferric iron was also measured and CRs based on weight loss and iron determination were in good agreement. Average CRs were 0.022 mm/yr for eight produced waters with high numbers (10^5^/ml) of acid-producing bacteria (APB), but no sulfate-reducing bacteria (SRB). Average CRs were 0.009 mm/yr for five central processing facility (CPF) waters, which had no APB or SRB due to weekly biocide treatment and 0.036 mm/yr for 2 CPF tank bottom sludges, which had high numbers of APB (10^6^/ml) and SRB (10^8^/ml). Hence, corrosion monitoring with carbon steel beads indicated that biocide treatment of CPF waters decreased the CR, except where biocide did not penetrate. The CR for incubations with 20 ml of a produced water decreased from 0.061 to 0.007 mm/yr when increasing the number of beads from 1 to 40. CRs determined with beads were higher than those with coupons, possibly also due to a higher weight of iron per unit volume used in incubations with coupons. Use of 1 ml syringe columns, containing carbon steel beads, and injected with 10 ml/day of SRB-containing medium for 256 days gave a CR of 0.11 mm/yr under flow conditions. The standard deviation of the distribution of residual bead weights, a measure for the unevenness of the corrosion, increased with increasing CR. The most heavily corroded beads showed significant pitting. Hence the use of uniformly sized carbon steel beads offers new opportunities for screening and monitoring of corrosion including determination of the distribution of corrosion rates, which allows estimation of the probability of high rate events that may lead to failure.

## Introduction

Corrosion of carbon steel is caused by physical, chemical, and microbiological factors (Beech and Sunner, 2004; Gieg et al., 2011; Enning and Garrelfs, 2014). It is a serious and expensive problem, which can lead for instance to failure of pipelines. When corrosion is uneven localized pits can form. These can be narrow and deep or shallow and wide (https://www.nace.org/Pitting-Corrosion/) and may eventually span the entire pipeline wall causing a failure. Because pitting corrosion is unpredictable pipelines must be regularly inspected to identify sections of decreased wall thickness, which may have experienced pitting corrosion. The surrounding areas may then be subjected to excavation to replace the corroded sections and prevent a future leak event. In the laboratory and in the field flat metal coupons are typically used to evaluate general and pitting corrosion by metal weight loss and by surface examination of the coupons, respectively. This allows definition of the pitting factor (P) as the ratio of the corrosion rate of the deepest pit (mm/yr) divided by the general corrosion rate (mm/yr).

The contribution of microbes to corrosion is often referred to as microbially-influenced corrosion (MIC). Although a wide variety of microbes may contribute the sulfate-reducing bacteria (SRB) are considered the main culprits. SRB can contribute to chemical MIC (CMIC) by reducing sulfate to sulfide using organic electron donors (organic acids, alcohols, or oil components), as shown for lactate in Equation 1. The produced sulfide can then react with carbon steel to form hydrogen (Equation 2), which is then also used by SRB as electron donor for sulfate reduction (Equation 3):

(1) $$\begin{array}{l} 2{\text{CH}}_{3}{\text{CHOHCOO}}^{-}+SO_{4}^{2-}+{\text{H}}^{+}\to 2{\text{CH}}_{3}{\text{COO}}^{-}\\ \;+2{\text{CO}}_{2}+{\text{HS}}^{-}+2{\text{H}}_{2}\text{O} \end{array}$$

(2) $$\begin{array}{l} {\text{H}}^{+}+{\text{HS}}^{-}+{\text{Fe}}^{0}\to {\text{FeS + H}}_{2} \end{array}$$

(3) $$\begin{array}{l} 4{\text{H}}_{2}+{\text{SO}}_{4}^{2-}+{\text{H}}^{+}\to {\text{HS}}^{-}+4{\text{H}}_{2}\text{O} \end{array}$$

Some SRB may be capable of directly using electrons derived from Fe^0^ (Equation 4) or using these through a H_2_ intermediate (Equations 3, 5):

(4) $$\begin{array}{l} 4{\text{Fe}}^{0}+{\text{SO}}_{4}^{2-}+9{\text{H}}^{+}\to {\text{HS}}^{-}+4{\text{Fe}}^{2+}+4{\text{H}}_{2}\text{O} \end{array}$$

(5) $$\begin{array}{l} 4{\text{Fe}}^{0}+8{\text{H}}^{+}\to 4{\text{Fe}}^{2+}+4{\text{H}}_{2} \end{array}$$

The latter is the classical mechanism through which SRB were thought to act (Von Wolzogen Kühr and Van der Vlugt, 1934). However, recent evidence has been presented that some SRB may be able to catalyze direct electron transfer from Fe^0^, as per Equation 4 (Dinh et al., 2004; Enning et al., 2012). This mechanism has been referred to as electrical MIC (EMIC). EMIC electron transport from Fe^0^ to cells may include electron-conductive corrosion products, e.g., FeS (Enning et al., 2012). Other hydrogenotrophic microbes, such as methanogens or acetogens may also contribute to MIC (Daniels et al., 1987; Dinh et al., 2004; Mand et al., 2014) through direct electron transfor or hydrogen intermediate mechanisms.

In studying the mechanism of corrosion or the corrosivity of field samples we have found that the use of carbon steel ball bearings (carbon steel beads) offers advantages for measuring general corrosion rates, because of their small size and therefore small weight variation. These beads can be used during incubations in serum bottles with or without shaking or can be packed in tubing or small columns for study of corrosion under flow conditions. Methods and results for these applications are presented here.

## Materials and methods

### Carbon steel beads and coupons

Beads of a36 carbon steel (Thomson Precision Ball, Rockwell hardness C60 to C67, Grade 200, ∅ = 3/32 = 0.0938 in; tolerance ± 0.001 in) were purchased from Motion Canada in Calgary, Alberta, Canada. Because of their use as ball bearings these are very similarly sized (55.0 ± 0.3 mg, ∅ = 0.238 ± 0.001 cm, A = 0.178 cm^2^). Coupons of ASTM a366 carbon steel (2.0 × 1.0 × 0.1 cm) were cut at the Engineering Workshop of the University of Calgary. Beads and coupons were pretreated according to National Association of Corrosion Engineers (NACE) protocol RP0775-2005. This involved briefly sanding the coupons with grit size 400 sand paper. Beads were rolled between two sheets of grit size 400 sand paper for 1–2 min. Coupons or beads were then treated for 2 min with inhibited HCl and then for 2 min with 1.2 M NaHCO_3_. The beads and coupons were washed with deionized water (dH_2_O), then with acetone and allowed to dry. Inhibited HCl was made by dissolving 10.6 g/L of dibutylthiourea in 37% (w/w) HCl and diluting with an equal volume of dH_2_O. The weight of the pretreated beads or coupons was then determined thrice and the average value was used as the starting weight. The combined weight of the pretreated beads was determined prior to incubation. Individual bead weights prior to incubation were assumed to be same as the average.

Following the use of beads or coupons in a corrosion experiment these were again treated with NACE protocol RP0775-2005 to remove adhering corrosion products. Following treatment with inhibited HCl and with NaHCO_3_ and washing with dH_2_O and with acetone, the dried beads were rolled between two Kimwipes (Kimtech Science Model S-8115) for 1 min to remove remaining loosely-associated corrosion product. The combined weight of coupons or beads was then determined thrice and the average used to calculate the weight loss (ΔW in g). The corrosion rate CR (mm/yr) was then calculated as:

(6) $$\begin{array}{l} \text{CR}=87,600\times \Delta \text{W}∕\left(\text{DxAxT}\right) \end{array}$$

Where D is the density of carbon steel (7.85 g/cm^3^), A is the surface area of the coupon or of the beads and T is the incubation time in h. The factor 87,600 converts the measured CR from cm/h to mm/yr. Weights of individual beads were also determined to characterize the change in weight distribution following corrosion.

### Physical, chemical, and microbial characterization of field samples

**Figure 1:**
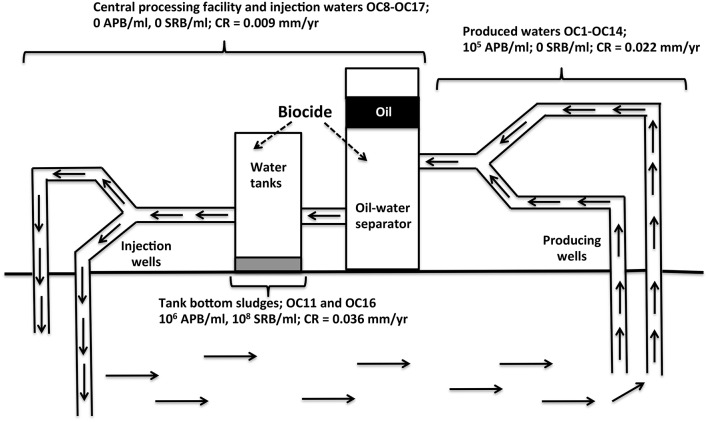
**Schematic of Oceania oil field from which samples OC1 to OC18 were obtained (Table 1)**. Samples of produced waters contained some oil, as these were collected upstream from the oil-water separator. Weekly biocide addition to water tanks of the central processing facility eliminated planktonic APB, but tank bottom sludges retained high numbers of APB and SRB and had the highest general CR.

Samples were obtained from an oil field in Oceania in December 2013 and January 2014 (2013/2014; Table 1) and in December 2014/January 2015 (2014/2015; Table 2). A schematic of the field indicating three distinct groups of samples is shown in Figure 1. The field has an *in situ* temperature of 70°C and produces light oil with an American Petroleum Institute (API) gravity of 40°. Samples of liquids were collected in 1 L glass or polyethylene containers, filled to the brim to exclude air as much as possible, whereas samples of solids were collected in Ziploc bags. Samples were stored and shipped at ambient temperature. Average transport time from the date of collection to arrival at the University of Calgary (UofC) was 2 weeks. Samples were stored in a Coy anaerobic hood in an atmosphere of 90% (v/v) N_2_ and 10% (v/v) CO_2_ (N_2_-CO_2_) immediately following arrival. This included samples of produced water (PW, which contained some produced oil), samples of central processing facility (CPF) water and of injection water (CPW and IW, which were oil free) and samples of solids and sludges (SS), retrieved from tank bottoms or from pipelines as pigging solids. Extracts from SS samples were made by contacting 15 g of SS with 15 ml of dH_2_O in the anaerobic hood, vortexing for 5 min and allowing the solids to settle by gravity. The supernatant was used as the SS extract.

**Table 1:** **Water chemistry, microbial numbers and corrosion rates of samples of produced water (PW), central processing facility water (CPW), injection water (IW), and solids and sludges (SS) obtained from an oil field in Oceania in 2013/2014**.

**Sample Name**	**Sample appearance**	**pH**	**NaCl (Meq)**	**Sulfate (mM)**	**Acetate (mM)**	**APB/ml^a^**	**SRB/ml^a^**	**CR (mm/yr) wt loss**	**CR (mm/yr) Fe diss**
OC1_PW	Clear water with oil	7.45	0.25	0.93	4.23	1.5 × 10^3^	<30	ND^b^	0.024
OC2_PW	Same as OC1	7.67	0.23	0.55	7.97	2.4 × 10^5^	<30	ND^b^	0.031
OC3_PW	Brown water with oil	5.81	0.49	1.77	4.44	4.3 × 10^3^	<30	0.032	0.027
OC4_PW	Same as OC1	6.51	0.25	0.58	13.19	4.3 × 10^6^	<30	0.024	0.029
OC5_PW	Same as OC1	7.11	0.3	0.87	6.82	2.4 × 10^7^	<30	0.014	0.024
OC6_PW	Same as OC1	7.4	0.17	0.74	5.68	<30	<30	0.022	0.024
OC7_PW	Same as OC1	7.75	0.1	1.01	2.11	4.3 × 10^5^	<30	0.026	0.015
OC12_PW	Same as OC1	7.47	0.18	0.82	5.22	4.3 × 10^5^	<30	0.017	0.02
OC13_PW	Same as OC1	7.46	0.21	0.68	4.51	2.4 × 10^6^	<30	0.025	0.024
OC14_PW	Same as OC1	7.35	0.2	0	4.34	4.3 × 10^3^	2.4 × 10^5^	0.017	0.014
	Average	7.2	0.24	0.8	5.85			0.022	0.023
	Standard deviation	0.6	0.1	0.44	3.03			0.006	0.006
OC8_CPW	Yellow water; no oil	6.54	0.18	6.29	67.67	<30	<30	0.003	0.005
OC9_IW	Clear water; no oil	6.92	0.15	2.45	33.98	<30	<30	0.005	0.008
OC10_IW	Same as OC8	6.92	0.2	2.72	52.15	<30	<30	0.003	0.01
OC18_CPW	Same as OC9	7.29	0.16	1.89	7.15	<30	<30	ND^b^	ND^b^
OC15_IW	Same as OC8	7.01	0.2	0.84	6.37	<30	<30	0.023	0.021
OC17_IW	Same as OC8	7.33	0.21	0.91	5.09	<30	<30	0.013	0.019
	Average	7	0.18	2.52	28.7			0.009	0.013
	Standard deviation	0.29	0.02	2	26.9			0.009	0.007
OC11_CPSS	Black oily solids	7.08	0	0	0.65	9.3 × 10^4^	2.4 × 10^8^	0.038	ND^b^
OC16_CPSS	Black oily solids	6.8	0	0	6.09	2.4 × 10^6^	4.3 × 10^7^	0.033	ND^b^
	Average	6.94	0	0	3.37			0.036	
	Standard deviation	0.2	0	0	3.85			0.004	

*CRs were determined with 20 carbon steel beads in a volume of 20 ml*.

a *MPNs of APB and SRB; lack of positive wells was scored as <30/ml because of the 0.1 ml volume used for 10-fold dilutions*.,

b *ND is not determined*.

**Table 2:** **Corrosion rates of carbon steel coupons and beads in serum bottles filled with samples of produced water (PW) or central processing facility water (CPW) at an Oceania oil field in 2014/2015^a^**.

**Sample**	**Carbon steel**	**Weight (g)**	**Back pressure (ml)**	**Corrosion rate (mm/yr)**
OC19_CPW	Coupons	2.654	24	0.018
OC20_CPW	Coupons	–	ND^b^	ND^b^
OC21_PW	Coupons	2.654	33	0.011
OC22_CPW	Coupons	2.654	51	0.014
OC19_CPW	Beads	0.276	37	0.122
OC20_CPW	Beads	0.276	48	0.089
OC21_PW	Beads	0.276	32	0.108
OC22_CPW	Beads	0.276	42	0.155

*CRs were determined for coupons or beads in a volume of 50 ml*.

a *Samples were shipped to the UofC and incubation was continued with shaking for a total of 45 days*.

b *Not determined as serum bottle broke during transport*.

Twenty one water samples (1 L) were also obtained from two fields in NE British Columbia and NW Alberta in which coiled tubing (CT) was used for removal of fracturing plugs during completion of horizontal wells for shale gas production operations.

Samples were subjected to general physical and chemical characterization. This included determination of the pH and of the salinity in molar equivalent of NaCl (Meq) with an Orion pH meter (model 370) and using the same meter with an Orion conductivity cell (model 013005MD), respectively. The concentration of dissolved sulfide was measured with the diamine method (Trüper and Schlegel, 1964). The concentrations of sulfate and acetate were determined by high performance liquid chromatography (HPLC). Sulfate was analyzed by ion chromatography using a conductivity detector (Waters 423) and IC-PAK anion column with borate/gluconate buffer at a flowrate of 2 ml/min (4 × 150 mm, Waters). Acetate was determined using an HPLC equipped with a UV detector (Waters 2487 Detector) and an organic acids column (Alltech, 250 × 4.6 mm) eluted with 25 mM KH_2_PO_4_ buffer at pH 2.5.

Most probable numbers (MPNs) of lactate-utilizing SRB and of glucose-fermenting acid-producing bacteria (APB) were determined from triplicate dilution series using 48-well microtiter plates (Shen and Voordouw, 2015). For MPNs of SRB, 0.1 ml of sample was inoculated into 0.9 ml of Postgate B medium, and then serially diluted 10-fold to 10^−8^ in the same medium in triplicate wells. The plate was immediately covered with a Titer-Tops membrane and incubated at 32°C inside the anaerobic hood. Wells were scored as positive when a black FeS precipitate was evident. For MPNs of APB, the sample was serially diluted in Phenol Red Dextrose medium (ZPRA-5, DALYNN Biologicals, Calgary, Alberta, Canada) using the same procedure as described for SRB. Growth of APB results in a color change of the medium from red to orange/yellow. MPN values were calculated by comparing the positive pattern to a probability table for triplicate MPN tests.

### Comparison of corrosion rates by weight loss and iron dissolution

In addition to measuring corrosion rate by weight loss, it was also measured as the release of ferrous iron measured spectrophotometrically with the ferrozine assay. Acid (1 N HCl) was used to dissolve ferrous iron from corrosion products, typically FeS and FeCO_3_, through a series of timed assays. Samples (20 ml) were placed in 50 ml serum bottles together with 20 acid pre-treated iron beads (1102 mg, 3.56 cm^2^). For samples OC11_SS and OC16_PS 20 ml of mixed solids, oil and water were used. Acid pretreatment was as per NACE protocol RP0775-2005. Sodium sulfide (20 μl of 1 M Na_2_S) was added and the serum bottles were then closed with butyl rubber stoppers. This procedure was done in a Forma anaerobic hood with an atmosphere of 85% N_2_, 10% CO_2_, and 5% H_2_. The samples were then incubated for 15–18 days at room temperature while lying flat on the platform of an orbital shaker, shaking at 150 rpm. At the end of the incubation each serum bottle was treated as follows. At *t* = 0 the stopper was removed, a stirring bar was added and a volume of 1.78 ml of 12 N HCl was injected, giving a final concentration of 1 N. At *t* = 1, 2 …10 min 50 μl of sample was removed and added to 450 μl of a solution of 0.7 M hydroxylamine in 1 N HCl and left to react for 15 min at room temperature. Following that 100 μl of this solution was added to 900 μl of ferrozine-HEPES solution at *t* = 16, 17, ….25 min incubated for a further 15 min after which the absorbance values were measured at 562 nm (A_562_) in 1 min intervals. The concentrations of dissolved iron were measured from a standard line for samples of ferric chloride in 1 N HCl, which were treated identically. The data (dissolved iron concentration vs. time, 1–10 min) were used to extrapolate the dissolved iron concentration at *t* = 0 and this value was used to calculate the weight of iron remaining at time zero (W_0_) and the general corrosion rate. The period from 10 to 15 min was used to remove the beads from the solution and washing these briefly with water, 1 M NaHCO_3_ (2 min) and then again briefly with water. They were then immersed in acetone (1 min) and placed on Kimwipes to dry. They were rolled briefly between Kimwipes to remove any remaining loose corrosion product. The beads were then weighed to determine weight loss. The determined weight loss for beads incubated in acid for 10 min (ΔW_10_) was corrected for weight loss during the 10 min acid treatment, by using the formula: ΔW_0_ = ΔW_10_ × C_Fe, 0_/C_Fe, 10_. The corrosion rate (mm/yr) was then calculated as CR = ΔW_0_/ATD, where A was the surface area of the beads (3.55 cm^2^), T was the incubation time and D was the density of iron (7.85 g/cm^3^).

### Corrosion rates of carbon steel beads or coupons inoculated in the field

Eight 120 ml serum bottles were sent to the field with either five carbon steel beads or two carbon steel coupons (2.0 × 1.0 × 0.1 cm) each. The beads and coupons were pre-treated as per NACE protocol RP0775-2005 and weighed. The bottles had an N_2_-CO_2_ headspace and contained 10 mg of sodium bisulfite as oxygen scavenger. Sterile plastic syringes (60 ml), needles, gloves and sampling instructions were sent together with the serum bottles to assure aseptic sampling. Field personnel added 50 ml of sample to each of the 120 ml serum bottles and these were sent to the UofC. Once received the samples were placed on a shaker at 30°C and incubated for a total of 45 days, including shipping time. At the end of the incubation, coupons and beads were treated with NACE protocol RP0775-2005.

### Microscopic examination

Following treatment with NACE protocol RP0775-2005, the carbon steel beads were viewed under a dissecting microscope. LED lights were used to illuminate the beads in order to increase contrast and improve detail. An Olympus SZ61 microscope fitted with an Infinity 2 Camera was used to take digital pictures of the carbon steel beads in various positions.

## Results

### Characteristics and corrosion rates of oceania oil field samples collected in 2013/2014

Samples were obtained from an oil field in Oceania from which oil was produced by water injection (Figure 1). Samples included produced waters, waters from the CPF, and tank bottom sludges (Figure 1, Table 1). Water tanks of the CPF were subjected to weekly biocide treatment to prevent MIC. Produced water samples collected in 2013/2014 (Table 1: OC1_PW to OC14_PW) had a near neutral pH (7.2), a low ionic strength (0.24 Meq of NaCl), a low sulfate (0.80 ± 0.44 mM), and a much higher acetate concentration (5.8 ± 3.0 mM). Water samples from the CPF and injection water samples (Table 1: OC8_CPW to OC17_IW) had a similar pH and ionic strength. However, these had higher concentrations of sulfate (2.5 ± 2.0 mM) and of acetate (28.7 ± 26.9 mM). Aqueous extracts of solids and sludges from tank bottoms of the CPF (Figure 1, Table 1: OC11_CPSS and OC16_CPSS) had a neutral pH, no salt, 0 mM sulfate, and 3.4 ± 3.8 mM acetate. All produced water samples, except OC6_PW, had a significant MPN of up to 2.4 × 10^7^ APB/ml. These samples had no significant MPNs of SRB with the exception of sample OC14_PW, which had 2.4 × 10^5^ SRB/ml. Water samples from the CPF and injection waters had no significant MPNs of APB or SRB. The elimination of planktonic APB from CPF and injection waters was likely the result of biocide addition to the CPF. However, this did not appear to affect the MPNs of extracts from solids and sludge samples OC11_CPSS and OC16_CPSS, which had 10^5^–10^6^ APB/ml and 10^7^–10^8^ SRB/ml (Table 1). This suggested that biocide did not effectively penetrate in tank bottom solids.

Corrosion rates (CRs) were measured by incubating 20 ml of sample with 20 pre-treated carbon steel beads (55.0 mg each) in 50 ml serum bottles under anaerobic conditions, while shaking at room temperature. The incubations were terminated by addition of 12 N HCl to a final concentration of 1 N. CRs were determined both from the measured concentration of dissolved and dissolving iron and from the measured weight loss, extrapolated to *t* = 0 (the time when acid was added). A typical spreadsheet for collection and calculation of corrosion data for sample OC6 is shown in Table S1. The dissolved iron concentration prior to acid treatment was measured to be 0.19 mM. Treatment with 1 N HCl gave an increase in dissolved iron with time as indicated in Table S1, giving an intercept of 3.03 mM. We interpret the intercept as due to the near instantaneous dissolution of corrosion products and the slope as due to the slower dissolution of metallic iron. Hence the actual concentration of Fe in corrosion product was 3.03–0.19 = 2.84 mM, corresponding to 3.25 mg in 20 ml and a CR of 0.024 mm/yr. Following incubation of iron beads in 1 HCl for 10 min, the beads were neutralized, washed, and dried. A weight loss of ΔW_10_ = 5.50 mg was measured at 10 min (Table S1). This was corrected for dissolution of metallic iron by noting that the concentrations of dissolved iron were 3.03 and 5.60 mM at 0 and 10 min, respectively. Hence, the corrected weight loss at 0 min was ΔW_0_ = 5.50 × 3.03/5.60 = 2.98 mg, which corresponded to a weight loss CR of 0.022 mm/yr. Hence CRs measured by iron dissolution and weight loss were in good agreement, as found for most other samples (Table 1). Poor agreement between the two methods to determine CR was observed when the concentration of dissolved iron in the sample could not be accurately determined, as for the two multi-phase sludges where large variations in dissolved iron were seen depending on whether aqueous or oily sub-samples were taken. We will, therefore, only refer to weight loss CRs in the results presented below.

The average weight loss CR for 8 produced waters was 0.022 ± 0.006 mm/yr, whereas that for 5 CPF and injection waters was lower at 0.009 ± 0.009 mm/yr (Table 1). The highest average CR was observed for the two solids samples at 0.036 ± 0.004 mm/yr. Hence, bacterial numbers and average CRs appeared to be related with highest CRs seen in the two solids and sludge samples which had high MPNs of both APB and SRB (Figure 1; Table 1).

### Effect of the number of carbon steel beads per unit volume on the measured corrosion rate

**Figure 2:**
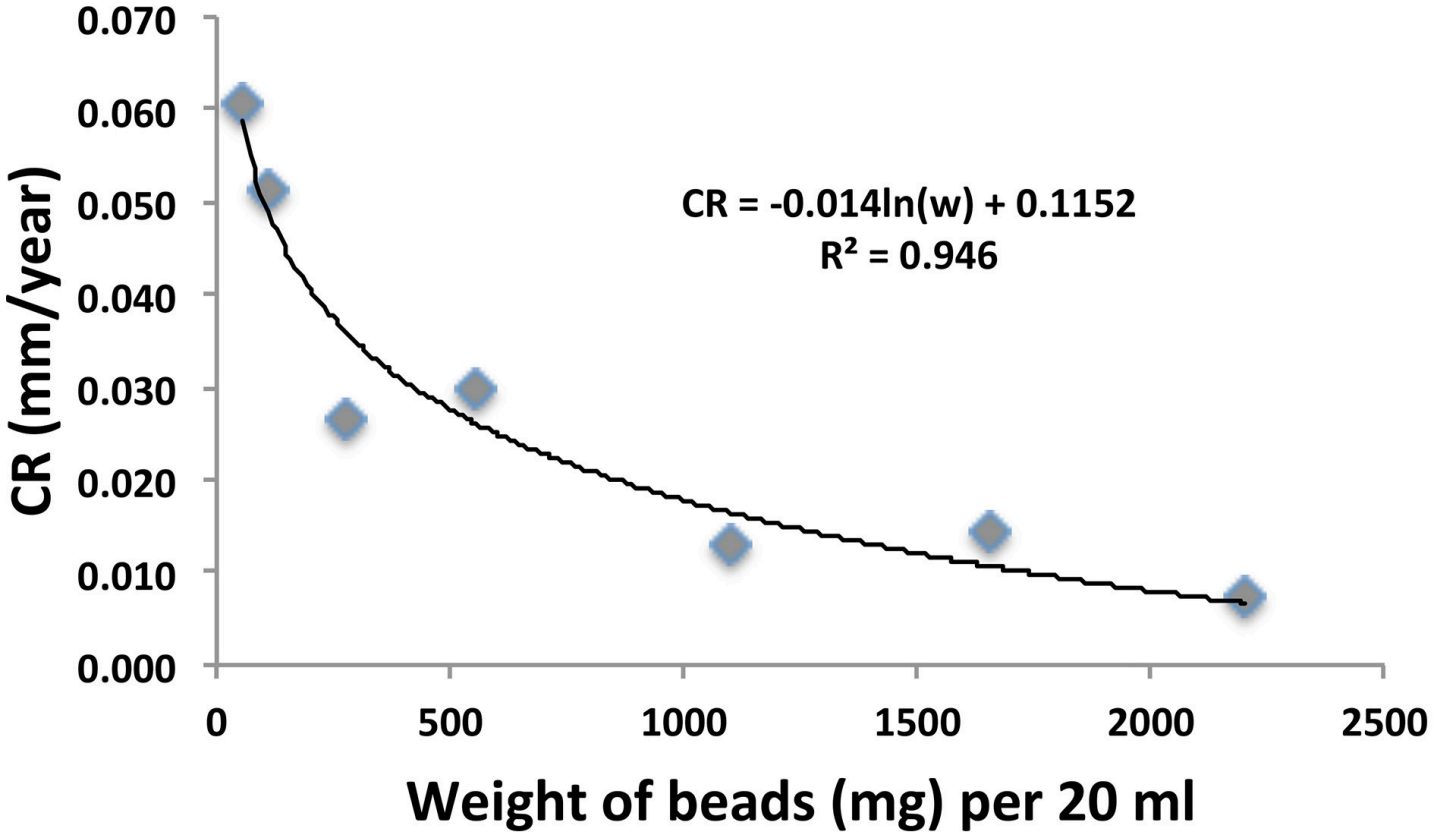
**Relation of corrosion rate (CR in mm/yr) and the weight of 55 mg carbon steel beads used in anaerobic incubations of sample OC2_PW in serum bottles, as described in the text**. The numbers of beads were from left to right 1, 2, 5, 10, 20, 30, and 40 in a constant volume of 20 ml.

Serum bottles containing 20 ml of sample OC2_PW and either 1, 2, 5, 10, 20, 30, or 40 carbon steel beads were incubated and corrosion rates were determined by weight loss. The corrected weight loss CRs obtained following treatment with 1 N HCl for 10 min are indicated in Figure 2. The results indicated that the measured CR decreased from 0.061 to 0.007 mm/yr, when the number of beads used in the analysis increased from 1 (55 mg of Fe) to 40 (2200 mg of Fe). Interestingly, the weight of carbon steel per unit volume is not routinely stated in corrosion studies. Having a larger weight of carbon steel in a small volume may stimulate the back reaction of the anodic dissolution of iron, decreasing the observed weight loss:

(7) $$\begin{array}{l} {\text{Fe}}^{2+}+2\text{e}\to {\text{Fe}}^{0} \end{array}$$

In view of the strong dependence of number of beads per unit volume on CR we have typically used only 5 beads per 20–50 ml. We also note that the decrease of CR with increased numbers of beads per unit volume (Figure 2) indicates that erosion corrosion from beads hitting each other during incubation with shaking did not contribute to the observed CR.

### Comparison of corrosion rates obtained with carbon steel beads and coupons

Capped and crimped serum bottles (120 ml) with either 2 coupons or 5 beads, 10 mg of sodium bisulfite and a headspace of N_2_-CO_2_ were shipped to the field, where they were filled with 50 ml sample. When the serum bottles were received at the UofC a backpressure of 32–51 ml was measured (Table 2), indicating that the crimped rubber stoppers provided a good seal. At the end of the 45-day incubation period the samples were pre-treated as per NACE protocol (RP0775-2005) and weighed to measure corrosion rates.

The average CR of six carbon steel coupons in three samples was 0.0142 mm/yr, whereas the average CR for 20 carbon steel beads (five in each of four samples) was 0.1185 mm/yr (Table 2). Hence the CRs measured with beads were on average eight-fold higher than those measured with coupons. Beads and coupons had a similar surface area of 3.24 and 2.80 cm^2^/g, respectively. This small difference was corrected for in the calculation of CR by Equation (6). A higher weight of carbon steel per unit volume for the incubation with coupons, as compared to beads (Table 2: 2.654 vs. 0.276 g) may have contributed to the decreased CR with coupons. Increased CRs with beads could be due to the fact that spherical beads make contact with the surrounding medium on all sides, whereas coupons (when not mounted in suspended coupon holders) tend to have a more exposed side (contacting the solution) and a less-exposed side (contacting the glass bottom of the serum bottles). Beads and coupons were made from a36 and a366 mild low carbon steel with carbon contents of 0.26 and 0.08% (w/w), respectively. These materials are similar, but not identical.

**Figure 3:**
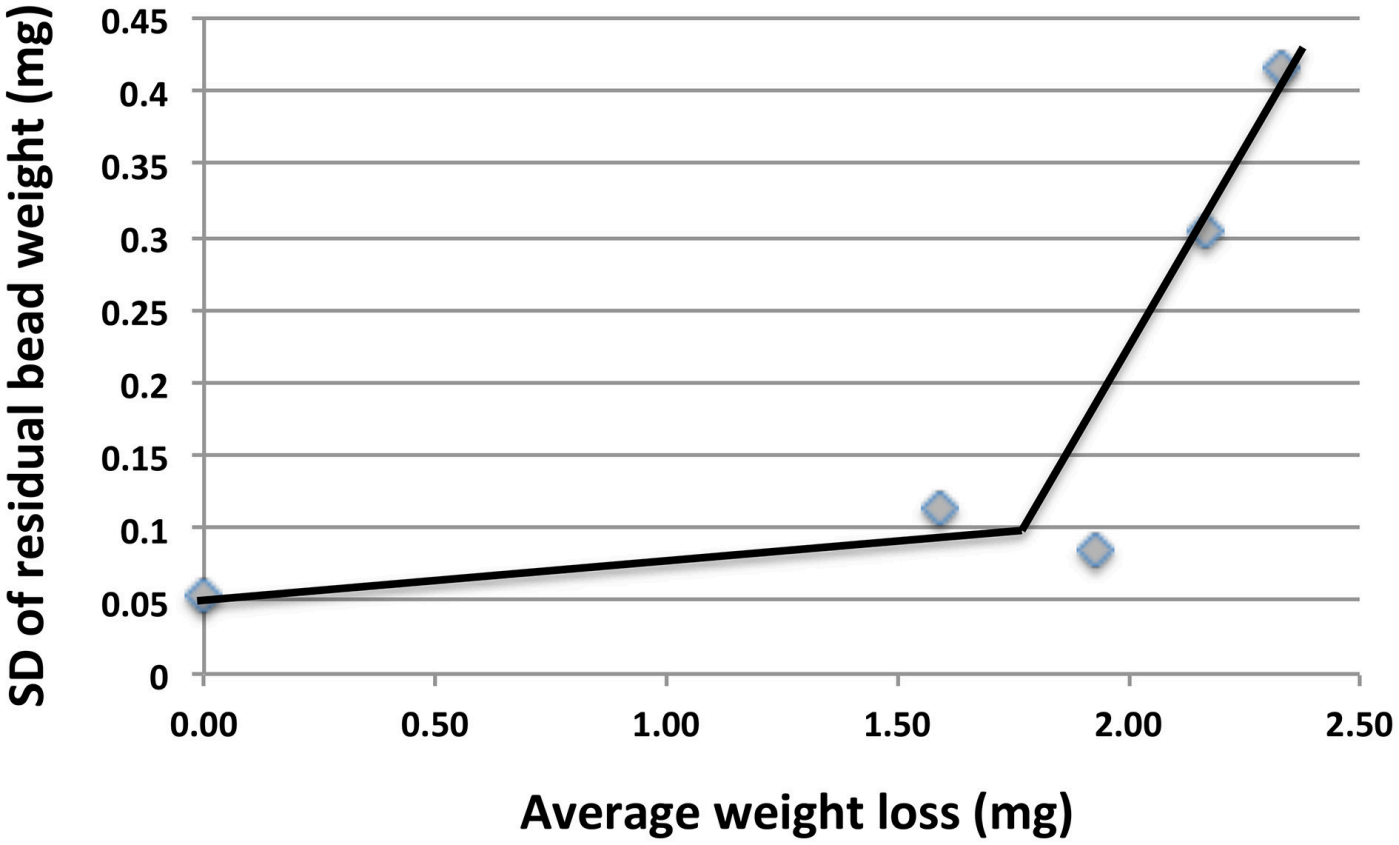
**Plot of standard deviation (SD) of residual bead weights vs. average weight loss for incubations in Table 2.** The increase in SD with increased weight loss indicates unevenness of the corrosion.

The CRs measured with beads in 2014/2015 samples (Table 2) were 5.4-fold higher than those measured in 2013/2014 samples (Table 1). This increase was partly caused by using a lower number of beads per unit volume (5/50 ml) in 2014/2015, than in 2013/2015 (20/20 ml). This increased the CR by an estimated 3.6-fold (Figure 2). Some of the difference may also have been caused by initiating corrosion experiments on site and by the longer incubation time of 45 days for the 2014/2015 samples. Interestingly we found that the standard deviation (SD in mg) of the average residual bead weight for the five beads present in incubations of 2014/2015 samples increased with decreasing average bead weight (Figure 3). This indicated the weight loss to become increasingly uneven as corrosion progressed.

**Figure 4:**
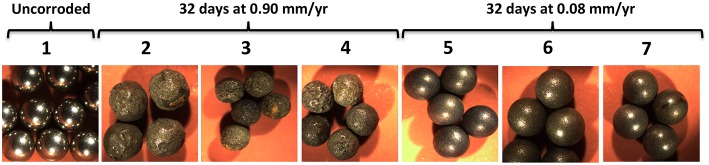
**Images of carbon steel beads (Ø = 0.238 cm)**. Photographs were taken of (1) pretreated beads prior to incubation and following exposure to (2–4) highly and (5–7) moderately corrosive conditions, as indicated.

Higher corrosion rates for beads as opposed to coupons were also observed with 21 samples obtained from coiled tubing (CT) operations in shale gas fields in NW British Columbia and NE Alberta. These samples had MPNs of APB, which varied from zero to 9.3 × 10^6^/ml, but no SRB, i.e., similar to the MPNs for most samples in Table 1. Samples (50 ml/120 ml serum bottle with an N_2_-CO_2_ head space) were incubated with either five beads for 39 days or with three coupons for 32 days. Coupons (1.3 × 0.8 × 0.4 cm) were cut from CT carbon steel. The average weight loss corrosion rate for all 21 samples was 0.054 ± 0.049 mm/yr (range 0.008–0.185 mm/yr) for coupons and 0.214 ± 0.261 mm/yr (range 0.09–1.31 mm/yr) for beads. Images of highly and moderately corroded beads are shown in Figure 4. Hence, the CRs measured with beads exceeded those measured with coupons by an average of 4.0-fold. These differences may be caused by systematic factors as indicated already. The CT samples also contained variable amounts of sand (0.4–8 g/L), used during fracturing, which may have contributed to erosion corrosion during incubation with shaking.

### Corrosion of carbon steel beads with acids

**Figure 5:**
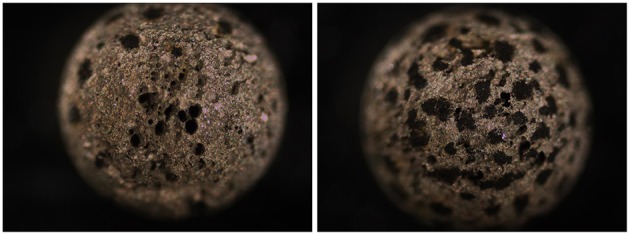
**Images of heavily corroded carbon steel beads subjected to 1 N of HCl for 51 h under anoxic conditions with shaking giving an average CR = 16.7 ± 3.0 mm/yr**. Extensive pitting is evident. Images of pretreated beads not subjected to incubation are shown in Figure 4.

Incubation of 50 pre-treated carbon steel beads in five 20 ml 1.5 × 15 cm Hungate tubes (10 beads/tube) each containing 15 ml of 1 N anoxic HCl and an N_2_-CO_2_ headspace for 51 h gave an average residual bead weight of 41.4 ± 8.9 mg (average ± SD for 50 beads), indicating highly uneven corrosion with an average CR of 16.7 ± 3.1 mm/yr. Indeed, extensive pitting was evident on the surface of highly corroded beads (Figure 5).

### Corrosion under flow conditions

Carbon steel beads (60, or 30 with 30 similarly sized glass beads) were packed in 1 ml syringe columns, which were subjected to injection of 10 ml/day of the effluent of a chemostat, containing actively growing SRB in defined medium with 10 mM lactate and 10 mM sulfate. The chemostat effluent had 10 mM acetate, 5 mM of residual sulfate, and 5 mM sulfide, indicating effective oxidation of lactate to acetate and CO_2_ and reduction of sulfate to sulfide as per Equation (1). The medium had excess sulfate allowing in principle reduction of sulfate with Fe^0^ as the electron donor in the syringe columns as per Equation (4). Up-flow injection of chemostat effluent into a carbon steel- and glass-bead containing column for 256 days gave an overall general corrosion rate of 0.11 mm/yr. The residual weight of the beads ranged from 36.5 to 51.4 mg, with an average of 44.0 ± 3.3 mg. The average weight of beads prior to corrosion was 55.0 ± 0.3 mg. The significant 11-fold increase in SD from 0.3 to 3.3 mg indicated uneven (pitting) corrosion. The most heavily corroded beads had deep pits (not shown). Short-term injection (46–60 days) of the same chemostat effluent into columns containing only carbon steel beads gave general corrosion rates of 0.016–0.030 mm/yr. We are currently trying to find conditions giving increased corrosion to allow rapid determination of the effect of treatment with biocides or corrosion inhibitors on corrosion under flow conditions.

## Discussion

The study of MIC involves determining (i) the contribution of microorganisms to general and pitting corrosion rates in the laboratory and in the field (ii) the structure and function of multiple species biofilms in catalyzing corrosion, and (iii) the mechanisms through which pure cultures or consortia of microorganisms are able to use metallic iron (Fe^0^) as electron donor for their metabolism.

Determination of CRs is typically done with coupons by electrochemical methods (Dexter et al., 1991) or by determining weight loss. The latter method is popular in MIC studies, where long incubation times are often required. As an example, Ilhan-Sungur et al. (2007) used galvanized carbon steel coupons (2 × 2 × 0.05 cm), covered with a small layer of zinc with the edges treated with epoxy primer. Twenty six coupons were exposed to a 1 L culture of the SRB *Desulfovibrio* sp. and the CR for eight sets of triplicate coupons was found to decrease from 0.035 to 0.001 mm/yr over 31 days. Rajala et al. (2015) incubated single carbon steel coupons (0.5 × 8 × 0.1 cm) with 250 ml samples of anoxic ground water from a radioactive waste storage site for 3–8 months and found CRs of 10^−3^–10^−4^ mm/yr. In contrast, when coupons were exposed to actual or simulated marine conditions, which included high sulfate concentrations and periodic exposure to air, much higher CRs of up to 1 mm/yr were observed (Enning et al., 2012; Marty et al., 2014). Marty et al. (2014) protected the coupon edges from corrosion by treatment with acrylic polyurethane to prevent edge effects. In our laboratory general weight loss CRs of coupons have been determined for field samples (as in this study), enrichments or cultures of pure strains (Park et al., 2011; Mand et al., 2014; Okoro et al., 2014).

General CRs have been classified by Al-Shamari et al. (2013) as low (<0.0254 mm/yr), moderate (0.0254–0.1245 mm/yr), high (0.127–0.254 m/yr), and severe (>0.254 mm/yr). Our results indicate that general CRs can be determined accurately with carbon steel beads over a wide range from 0.001 to 10 mm/yr. The absence of edge effects and the fact that beads have no preferred side of contact with glass during incubation with shaking are advantages over the use of coupons. Determination of precise general CRs allows assessment whether biocide addition decreases general CRs (Figure 1, Table 1). Because higher CRs are measured when less carbon steel is added to a corrosion assay (Figure 2), it is important that the weight of carbon steel added per unit volume is standardized. It is of interest in this regard that high weight loss CRs (0.70 mm/yr) catalyzed by marine EMIC-catalyzing SRB were achieved by incubating a 10 × 10 × 1 mm coupon (~785 mg of iron) in 1.4 L of medium (Enning et al., 2012). At 11 mg/20 ml this is 1/5 of the lowest value in Figure 2 (1 bead of 55 mg/20 ml). Using the fitted curve in Figure 2 we estimate that CRs in Table 1 increase by about five-fold at such a low weight per unit volume with the highest value observed (Table 1: 0.038 mm/yr) increasing to 0.190 mm/yr. Hence, standardization of weight per unit volume is important to allow other factors, which may cause differences in CR, such as surface to volume ratio and type of carbon steel to be assessed.

The flat surfaces of coupons can be examined for pitting corrosion by optical microscopy to find the deepest pit (Johnston and Voordouw, 2012), by scanning electron microscopy (SEM) of fixed, dehydrated coupons (Nemati et al., 2001; Hubert et al., 2005; Enning et al., 2012), or by SEM- or optical microscopy-mediated profilometry (Liang et al., 2014). SEM-associated methodologies allow analysis of corrosion product composition. The biofilm formed on coupons has been analyzed by sequencing of PCR-amplified 16S rRNA genes, fluorescence *in situ* hybridization (FISH) or other methods (Zhang et al., 2003; Zhu et al., 2003; Rajala et al., 2015). Profilometry of spherical beads is clearly difficult, as this requires rotation to be able to scan the entire surface. However, limited sections of the surface can be seen (Figure 5) and analyzed, as has been done for corroded ball bearings (Squires and Radcliffe, 1983). A unique feature of the homogeneous size of beads is that it allows measurement of the corrosion of multiple simultaneously incubated specimens. Increasing unevenness of the corrosion can then be evaluated as the increase in SD of the weight distribution of multiple beads (Figure 3). The most heavily corroded beads in this distribution tend to have significant pits or other surface features (Figures 4, 5). The microbial community composition of adherent biofilms can be evaluated by transferring beads into microfuge tubes and proceeding directly with DNA isolation.

The study of the effective use of metallic iron (Fe^0^) as electron donor for microbial metabolism is facilitated by using forms of iron with a large surface-to-volume ratio and a reactive surface. Early studies on the co-metabolism of Fe^0^ by SRB growing on lactate and sulfate used steel wool as the substrate with a surface to volume ratio of 100 cm^3^/g (Cord-Ruwisch and Widdel, 1986). Likewise an early demonstration of Fe^0^ corrosion by methanogens used iron powder of which the properties were not stated (Daniels et al., 1987). More recent studies on the isolation and characterization of SRB, methanogens and acetogens, which use Fe^0^ effectively as electron donor for the reduction of sulfate to sulfide, of CO_2_ to methane or of CO_2_ to acetate, have made use of Fe^0^ granules with a diameter of 0.1–0.2 cm (Dinh et al., 2004; Mori et al., 2010; Uchiyama et al., 2010; Kato et al., 2014). The rate of anodic dissolution of ferrous iron was typically reported in these experiments, but the CR was not. The use of homogeneously sized carbon steel beads in such studies would allow calculations of precise CRs and an initial evaluation of the potential for pitting corrosion from evaluation of the increase in SD of the weight distribution. Using beads in culturing may allow the identification of surface activity, which cannot be evaluated with less defined powders or granules. Highly pitted carbon steel beads, which may serve as an even better substrate for microbial growth, can be easily generated by prolonged acid treatment (Figure 5).

In conclusion, the use of carbon steel beads offers additional opportunities for screening and monitoring of corrosion, including MIC, in oil field samples. This includes determination of the distribution of corrosion rates, characterized by a mean and an SD allowing estimation of the probability of occurrence of rates high enough to lead to failure.

## Author contributions

GV—planned experiments, monitored progress and suggested revisions to protocols, drafted the final manuscript. PM—water chemistry analysis of oilfield samples from Oceania, conducted corrosion experiments with these samples. TP—conducted the carbon bead experiments in upflow bioreactors and associated water analysis and corrosion rate measurements. MS—did experiments with coiled tubing samples including water analysis, corrosion experiments, and data interpretation. YS—conducted all MPN assays for all samples in the study. AV—did experiments on corrosion of carbon steel beads in varied concentrations of acids. JV—assisted with upflow bioreactor carbon bead experiments and acid experiments with carbon steel beads. AS—coordinated collection and shipment of Oceania field samples to the lab. Provided facility operational details to assist with data interpretation. Contributed to manuscript preparation.

### Conflict of interest statement

The authors declare that the research was conducted in the absence of any commercial or financial relationships that could be construed as a potential conflict of interest.
