# Supplementary material for: Use of Homogeneously-Sized Carbon Steel Ball Bearings to Study Microbially-Influenced Corrosion in Oil Field Samples
Source: Front Microbiol. 2016 Mar 24;7:351. doi: 10.3389/fmicb.2016.00351 (PMC4805590; doi:10.3389/fmicb.2016.00351)
Supplement: Supplementary file 1 [file Table1.DOCX]

frontiers

**Table S1.** Excel spreadsheet for analysis of corrosion data for sample OC6. Cferric (mM) is plotted against time (min) and the intercept (3.033 mM) is used to calculate the corrosion rate, based on iron dissolution. The weight loss at 10 min (5.5 mg) is corrected for acid dissolution of metallic iron using the data in the chart to 2.98 mg. The derived corrosion rates (bold) are indicated in Table 1.

| | OC6 | Start date 20-12-2013 | | Date analyzed: 7-1-2014 | | 18 days | 432 h | |
|---|---|---|---|---|---|---|---|---|
| | T (days) | sample (ul) | V (ul)HCl-NH | I (ul) | Vfeoz (ul) | A562 | A*D | Cferric (mM) |
| Fe before | 0 | 50 | 450 | 100 | 900 | 0.052 | 5.2 | 0.19 |

| | T (min) | sample (ul) | V (ul)HCl-NH | I (ul) | Vfeoz (ul) | A562 | A*D | Cferric (mM) |
|---|---|---|---|---|---|---|---|---|
| | 1 | 50 | 450 | 100 | 900 | 0.717 | 71.7 | 3.20 |
| | 2 | 50 | 450 | 100 | 900 | 0.795 | 79.5 | 3.55 |
| | 3 | 50 | 450 | 100 | 900 | 0.858 | 85.8 | 3.83 |
| | 4 | 50 | 450 | 100 | 900 | 0.911 | 91.1 | 4.07 |
| | 5 | 50 | 450 | 100 | 900 | 0.954 | 95.4 | 4.27 |
| | 6 | 50 | 450 | 100 | 900 | 1.067 | 106.7 | 4.78 |
| | 7 | 50 | 450 | 100 | 900 | 1.068 | 106.8 | 4.79 |
| | 8 | 50 | 450 | 100 | 900 | 1.139 | 113.9 | 5.11 |
| | 9 | 50 | 450 | 100 | 900 | 1.184 | 118.4 | 5.31 |
| | 10 | 50 | 450 | 100 | 900 | 1.232 | 123.2 | 5.53 |
| Total corroded (mg): | | | 3.25 | | | intercept | 3.0333 | 2.85 |
| Total Fe added, 20 beads (mg) | | | 1102.60 | | | | | |
| A (cm2) | | | 3.55 | | | | | |
| T (hr) | | | 432.00 | | | | | |
| **CR (mm/yr)** | | | **0.024** | | | | | |
| Beads after 10 min of HCl, NH2OH | | | 1097.1 | | | | | |
| Total corroded (mg, wt loss at 10 min) | | | 5.50 | | | | | |
| 0.2565 | 3.0333 | 5.5983 | 2.98 | | | | | |
| **CR (mm/yr), wt loss** | | | **0.022** | | | | | |

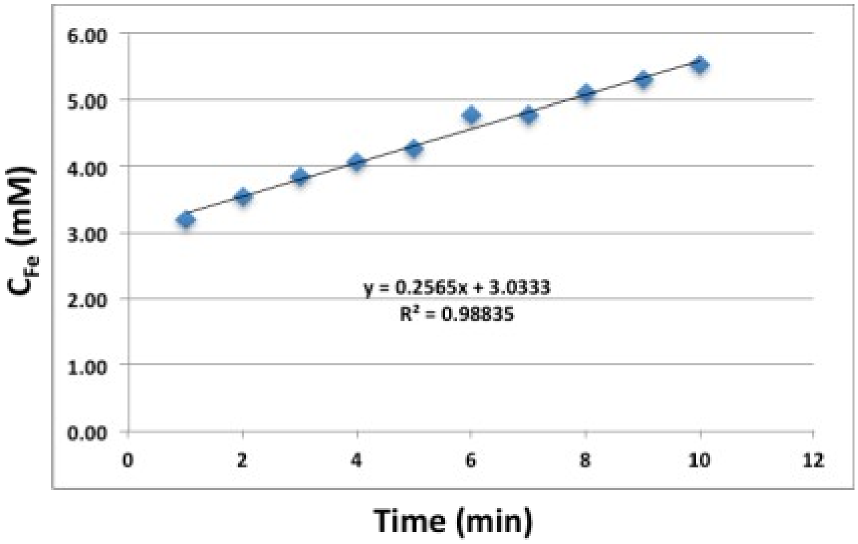


1
